# The future of biomarkers for vascular contributions to cognitive impairment and dementia (VCID): proceedings of the 2025 annual workshop of the Albert research institute for white matter and cognition

**DOI:** 10.1007/s11357-025-01746-y

**Published:** 2025-06-21

**Authors:** Matthew J Lennon, Nikolaos Karvelas, Aravind Ganesh, Shawn Whitehead, Farzaneh A Sorond, Violeta Durán Laforet, Elizabeth Head, Konstantinos Arfanakis, Vijaya B Kolachalama, Xiao Liu, Hanzhang Lu, Joel Ramirez, Keenan Walker, Erica Weekman, Cheryl L Wellington, Charisse Winston, Frank C Barone, Roderick A Corriveau

**Affiliations:** 1 Centre for Healthy Brain Aging (CHeBA), Discipline of Psychiatry & Mental Health, School of Clinical Medicine, University of New South Wales, Sydney NSW, Australia; 2 Northern Sydney Local Health District, NSW Health, Sydney NSW, Australia; 3 Departments of Neurology and Neuroscience, Icahn School of Medicine at Mount Sinai, New York, NY USA; 4 Department of Clinical Neurosciences and Hotchkiss Brain Institute, Cumming School of Medicine, University of Calgary, Calgary, AB Canada; 5 Department of Anatomy and Cell Biology, Schulich School of Medicine and Dentistry, Western University, London, ON N6A5C1 Canada; 6 Department of Neurology, Division of Stroke and Neurocritical Care, Northwestern University Feinberg School of Medicine, Chicago, IL USA; 7 Department of Neurobiology, Brudnick Neuropsychiatric Research Institute, University of Massachusetts Chan Medical School, Worcester, MA USA; 8 Department of Pathology & Laboratory Medicine, University of California, Irvine, CA 92697 USA; 9 Department of Biomedical Engineering, Illinois Institute of Technology, Chicago, IL USA; 10 Department of Diagnostic Radiology and Nuclear Medicine, Rush University Medical Center, Chicago, IL USA; 11 Rush Alzheimer’s Disease Center, Rush University Medical Center, Chicago, IL USA; 12 Department of Medicine, Boston University Chobanian & Avedisian School of Medicine, Boston, MA 02118 USA; 13 Department of Computer Science and Faculty of Computing & Data Sciences, Boston University, Boston, MA 02215 USA; 14 Department of Biomedical Engineering, The Pennsylvania State University, University Park, PA 16802 USA; 15 Institute for Computational and Data Sciences, The Pennsylvania State University, University Park, PA 16802 USA; 16 Department of Radiology, Johns Hopkins University, School of Medicine, Baltimore, MD USA; 17 Sunnybrook Research Institute, University of Toronto, Toronto, ON Canada; 18 Laboratory of Behavioral Neuroscience, National Institute on Aging, National Institutes of Health, Baltimore, MD USA; 19 Stark Neurosciences Research Institute, Indiana University School of Medicine, Indianapolis, IN USA; 20 Sanders-Brown Center on Aging, University of Kentucky, Lexington, KY USA; 21 Department of Pathology and Laboratory Medicine, Djavad Mowafaghian Centre for Brain Health, University of British Columbia, Vancouver, BC Canada; 22 University of Southern California, Alzheimer′s Disease Therapeutic Research (ATRI), San Diego, CA USA; 23 University of California, La Jolla, San Diego, CA USA; 24 Department of Neurology, University of New York Downstate Health Sciences University, Brooklyn, NY USA; 25 Division of Neuroscience, National Institute of Neurological Disorder and Stroke (NINDS), Bethesda, MD USA

**Keywords:** Biomarkers, Vascular contributions to cognitive impairment and dementia, Cognition

## Abstract

Advances in biomarkers and pathophysiology of vascular contributions to cognitive impairment and dementia (VCID) are expected to bring greater mechanistic insights, more targeted treatments, and potentially disease-modifying therapies. The 2025 Annual Workshop of the Albert Research Institute for White Matter and Cognition, sponsored by the Leo and Anne Albert Charitable Trust since 2015, focused on novel biomarkers for VCID. The meeting highlighted the complexity of dementia, emphasizing that the majority of cases involve multiple brain pathologies, with vascular pathology typically present. Potential novel approaches to diagnosis of disease processes and progression that may result in VCID included measures of microglial senescence and retinal changes, as well as artificial intelligence (AI) integration of multimodal datasets. Proteomic studies identified plasma proteins associated with cerebral autosomal dominant arteriopathy with subcortical infarcts and leukoencephalopathy (CADASIL; a rare genetic disorder affecting brain vessels) and age-related vascular pathology that suggested potential therapeutic targets. Blood-based microglial and brain-derived extracellular vesicles are promising tools for early detection of brain inflammation and other changes that have been associated with cognitive decline. Imaging measures of blood perfusion, oxygen extraction, and cerebrospinal fluid (CSF) flow were discussed as potential VCID biomarkers, in part because of correlations with classic pathological Alzheimer’s disease (AD) biomarkers. MRI-visible perivascular spaces, which may be a novel imaging biomarker of sleep-driven glymphatic waste clearance dysfunction, are associated with vascular risk factors, lower cognitive function, and various brain pathologies including Alzheimer’s, Parkinson’s and cerebral amyloid angiopathy (CAA). People with Down syndrome are at high risk for dementia. Individuals with Down syndrome who develop dementia almost universally experience mixed brain pathologies, with AD pathology and cerebrovascular pathology being the most common. This follows the pattern in the general population where mixed pathologies are also predominant in the brains of people clinically diagnosed with dementia, including AD dementia. Intimate partner violence-related brain injury, hypertension’s impact on dementia risk, and the promise of remote ischemic conditioning for treating VCID were additional themes.

## Introduction

The science of vascular contributions to cognitive impairment and dementia (VCID) is a field of research investigating the hypothesis that significant dementia-related cognitive decline results from damage to brain function due to vascular injuries of any type [1]. Diagnoses that fall within the VCID scientific research framework are numerous and include common syndromes such as Alzheimer’s disease (AD) dementia and vascular dementia, as well as rare diagnoses with a proven genetic cause, such as CADASIL. Examples of other relevant diagnoses with hypothesized vascular causes and contributions to dementia include vascular cognitive impairment, multi-infarct dementia, post-stroke dementia, cerebral amyloid angiopathy, and Binswanger’s disease.

Dementia is a clinically defined construct typically considered the presence of significant cognitive impairment and functional decline that interferes with activities of daily living. Numerous clinical diagnoses fall under the dementia umbrella, with Alzheimer’s disease dementia (ADD) being the most common. It is notable that clinically diagnosed ADD, in the absence of pathological data, is typically referred to simply as AD. The inverse is also true. When AD pathology is present, including in the absence of clinical data, a diagnosis of AD is typically assigned. For this reason, and for scientific clarity, when possible, we refer to ADD and pathological AD instead of using AD as a standalone term. Widely used dementia diagnoses also include frontotemporal dementias, Lewy body dementias, vascular dementias, and others. Over the past two decades, it has become clear that, in most cases, a clinically assigned dementia diagnosis only rarely corresponds to a pathologically “pure” brain state [2]. Rather, it is typical for more than one dementia-associated pathology to be present in the brain of an individual with cognitive decline. Moreover, it has also been shown that, in a small but tangible number of cases, the anticipated brain pathology based on a clinically assigned diagnosis does not match the post-mortem pathological diagnosis. This may not be surprising, however, because unless a rare autosomal dominant genetic cause of dementia is present, dementia diagnoses are syndromic, which can lead to a mistaken sense of “knowing the cause” when, more often than not, the root cause is not strictly known. Nonetheless, it is rigorously established that most cases of dementia are associated with some type of aggregated protein as well as, very commonly, vascular pathology.

Thus, it is critical to have a better understanding of cerebrovascular contributors to dementia along with the urgent need for biomarkers for the broad scope of potential causes and risk factors for dementia. The focus of this meeting was on biomarkers for VCID [1, 3], with an emphasis on identifying VCID where it occurs rather than being confined by traditional clinical diagnostic boundaries. This is critical because it is increasingly reported that dementia risk increases as the number of brain pathologies increases, e.g., coexisting AD pathology and vascular pathology nearly doubles the risk of clinical dementia compared to AD pathology alone [4].

The 2025 Annual Workshop of the Albert Research Institute for White Matter and Cognition brought together a diverse range of subspecialty experts to consider the challenges of VCID classification, pathophysiology, and treatment. The workshop had a particular focus on novel biomarker identification, recognizing the crucial role they play in understanding disease pathogenesis, making diagnoses, monitoring treatment outcomes, and stimulating further understanding and investment into disease management.

## Summary of 6–8 January 2025—6th annual Albert research institute for white matter and cognition workshop

The 2025 workshop scientific program was organized into four topics: (i) novel approaches to dementia biomarkers, (ii) fluid biomarkers, (iii) imaging biomarkers, and (iiii) risk and disease management. The meeting was held in person in Clearwater, Florida.

As an introduction to the workshop, Dr. Roderick A. Corriveau, Director of the National Institutes of Neurological Disorders and Stroke (NINDS) Vascular Contributions to Cognitive Impairment and Dementia (VCID) portfolio, presented the rationale behind the focus of the program on vascular pathologies as mediators of cognitive decline. Post-mortem analyses have demonstrated that most common dementia cases, regardless of clinically assigned diagnosis, have multiple brain pathologies that are hypothesized to result in cognitive decline. For this reason, the National Plan to Address Alzheimer’s Disease includes prioritization of multiple etiology dementias (MED). Because most dementia cases are MED, and because brain vascular pathology is present in most MED cases, the NINDS is advancing the science of VCID. In brief, the science of VCID focuses on testing the hypothesis that vascular pathologies contribute to, and in some instances are the root cause of, cognitive decline and dementia [1]. This hypothesis is being tested in more than 150 active investigator-initiated NIH awards, as well as research funded under 21 NINDS-led VCID targeted funding announcements including four major NINDS programs: MarkVCID, DiverseVCID, DISCOVERY, and the VCID Center Without Walls. The NINDS VCID portfolio aims to promote rigorous approaches to determine clinical consequences of vascular pathologies associated with dementia, interactions with other pathologies, and develop evidence-based understanding of which molecular, cellular, and physiological mechanisms cause or do not cause cognitive decline. Knowledge resulting from these programs should position the field to move toward more effective biomarkers, targets, and treatments to lessen the burden of dementia. A critical goal going forward is to further develop VCID biomarkers at all levels from early stage discovery to instrumental validation, clinical validation, and with rigorously established biomarker category and context of use in each instance, following FDA guidance with the goal of achieving agency approval and widespread use in clinical trials and, ultimately, everyday clinics. The introductory remarks by Dr. Corriveau offered a unified framework under which the different avenues of work, presented in the following sessions, coalesce to build our understanding of VCID.

### Novel approaches to dementia biomarkers

The clinical diagnosis of vascular dementia poses a challenging task, owing to the differences in diagnostic criteria and the heterogeneity of underlying mechanisms. Previous diagnostic criteria for vascular dementia have involved the establishment of cognitive impairment, sometimes exhibiting specific patterns, coexisting with cerebrovascular disorders/pathology, as identified through history, clinical examination, or imaging [5, 6]. However, the above criteria rely on the co-occurrence of pathophysiological processes, which might be incidental and clinically silent, while also overlooking the potential synergistic effects of vascular pathologies with proteinopathies and other mechanisms. Accordingly, there is a growing need for the development of novel, specific, or etiologic markers for VCID. The first session of the workshop examined such avenues of work. The conference covered a select few target novel biomarkers. A more comprehensive review of potential future biomarkers has been published by Cipollini et al. [7].

Spatial omics technologies enable an unprecedented view at the subcellular level of disease. Potential biomarkers can be identified based on their direct spatial relation to pathologies of interest. As the inaugural speaker of this session, Dr. Violeta Durán Laforet presented her work exploring the connection between senescence and inflammation in neurodegeneration through spatial transcriptomics. Cellular senescence is emerging as a key feature across several neurodegenerative diseases, with recent evidence pointing towards microglial senescence as a key mechanism [8]. Dr. Durán Laforet employed multiplexed error-robust fluorescence in situ hybridization (MERFISH) [9] to provide spatial maps of cellular senescence signatures in physiological aging and AD mouse models, based on comprehensive expression of a cassette of universal senescence-related genes. Glial cells emerged as the most pronounced senescent cell group, a finding validated in human AD dementia and shown to be exacerbated with disease severity. Through senescent microglia profiling, a microglia-derived senescence associated secretory factor, CXCL3, that drives AD dementia-relevant behavioral alterations in mouse models was identified. This work provided both mechanistic insight for the involvement of microglial senescence in neurodegeneration and provided an example of how spatial omics technologies can be implemented to derive novel diagnostic and therapeutic targets.

Currently, magnetic resonance imaging (MRI) is the most widely used technique to assess the presence, extent, location, and type of vascular lesions in cerebral small vessel disease (cSVD). However, MRI-based biomarkers still lack specificity and sensitivity for VCID, while also being costly, thus precluding regular follow-up of patients. Retinal imaging has emerged as an alternative in recent years, having the advantage of direct visualization of cerebrovascular arteries. Dr. Erica M. Weekman’s work focuses on understanding the mechanisms behind the retinal changes observed in human pathological AD and cSVD that reflect brain disease. Using a mouse model of hyperhomocysteinemia (HHcy)-induced cSVD, her group aimed to identify the effects of cSVD on visual sensitivity and cognition, retinal glial and vascular cells, and neuroinflammatory and cardiovascular gene expression changes. Ultimately, HHcy led to visual deficits that specifically affected the reaction to blue and white light, decreased vascular volume, and decreased interaction of microglia with the vasculature, as well as downregulation of inflammatory and vascular genes. These results highlight retinal changes in association with cSVD, offering preclinical evidence for their translation to human biomarkers, and serve as a precaution when interpreting vision-dependent cognitive testing in cSVD models.

As discussed during the meeting, despite challenges associated with current diagnostic methods, they still provide valuable information, some of which may be imperceptible to human evaluation. Dr. Vijaya B. Kolachalama’s research highlighted that Artificial intelligence (AI) has the potential to process vast amounts of data and uncover previously unknown interconnections, particularly in distinguishing neurodegenerative disease patterns. AI-driven machine learning frameworks can integrate multimodal data, including neuroimaging, cognitive assessments, and clinical histories, to improve differentiation between Alzheimer’s disease and non-Alzheimer’s dementias [10]. Expanding on this, AI models have been applied to vascular dementia, detecting early cerebrovascular changes and analyzing cognitive profiles [11]. These advancements highlight AI’s transformative potential in enhancing the differential diagnosis of dementia, improving diagnostic accuracy, and ultimately supporting better patient care [12].

Overall, the initial session showcased state-of-the-art lines of work, with transformative potential for the VCID and dementia fields. Rapid advances in biomedical technology enable expansion of available tools for diagnosis and prognostication of VCID.

#### Fluid biomarkers

Markers of disease in biological fluids, including but not restricted to plasma and cerebrospinal fluid (CSF), have been gaining traction as accurate and accessible predictors of dementia, particularly AD dementia. In the case of VCID, discovery of pathophysiology-driven markers has been challenging, owing to the non-specificity of mechanisms, such as endothelial activation and hypercoagulability, to cerebrovascular injury [13]. The discovery efforts for specific and highly predictive biofluid VCID markers was the main focus of this session.

The plasma proteome offers an attractive choice for molecular marker discovery, as it can reflect both brain and vascular disease. Currently, little is known about the biological processes and peripherally circulating molecular mediators that directly influence cSVD development and related phenotypes. Dr. Keenan A. Walker presented results connecting clinical and imaging phenotypes of VCID with molecular perturbations in the plasma. Using a large-scale proteomic platform to relate 4877 plasma proteins to MRI-defined SVD characteristics and VCID phenotypes, his group identified proteins associated with white matter hyperintensity (WMH) volume, motoric cognitive risk, and vascular dementia [14, 15]. These relationships were replicated in one or more external cohorts and demonstrated that several of these proteins are primarily expressed by oligodendrocytes, whereas many others were found to play a role in metabolic and immune processes. A subset of these proteins was also found to be differentially expressed in the context of cerebral atherosclerosis and pathologically defined AD in publicly available datasets, providing evidence of connection between vascular and neurodegenerative disease processes. Finally, his group causally implicated a protein network enriched for complement activation in WMH development using two sample Mendelian randomization, and prioritized two plasma proteins, oligodendrocyte myelin glycoprotein (OMG) and neuronal pentraxin receptor (NPTXR), as candidate biomarkers for cSVD, white matter disease, and related clinical manifestations.

The heterogeneity of underlying causes and manifestations of VCID, however, can complicate the detection of plausible markers. Monogenic forms of VCID offer a unique opportunity for the discovery of mechanisms and biomarkers that could translate to sporadic VCID. Cerebral autosomal dominant arteriopathy with subcortical infarcts and leukoencephalopathy (CADASIL) is a monogenic form of cSVD) due to mutations in the *NOTCH3* gene. CADASIL presents with early white matter injury, eventually leading to strokes and VCID after the 4th decade of life [16]. The known clinical trajectory of CADASIL allows for the identification of proteomic changes coinciding with the phenotypic transition from cSVD to VCID. Dr. Nikolaos Karvelas presented his findings on prognostic proteomic markers in plasma from CADASIL patients. His previous work utilizing proteomics had found robust plasma signatures of disease through machine learning methods that suggest dysregulations in fibrosis, angiogenesis, and metabolism as core features of CADASIL [17]. He had also previously shown correlations of perivascular space enlargement with white matter injury, cognitive decline, and proteomic markers of endothelial activation and inflammation [18], thereby supporting potential prognostic markers. Participants with CADASIL and healthy controls were pooled from three distinct cohorts (VascBrain, Mayo Clinic, Columbia-Boston) and categorized into three major clinical stages based on the natural history of CADASIL: asymptomatic/early symptomatic (migraine), stroke without cognitive impairment, and cognitive impairment. Differential expression analysis (DEA) and weighted protein co-expression network analysis (WPCNA) were performed to derive protein signatures. Upregulation of muscle cell and inflammatory markers was evident in stroke, suggesting mural cell loss. In cognitive impairment, an increase in cell-mediated immunity markers and downregulation of metabolic processes were noted. Finally, a network of mural cell loss was associated with the clinical stage of progression and also with a network of fibrosis-coagulation with age only in CADASIL, indicating the central role of these processes in disease progression. These results suggest that blood proteomic signatures of CADASIL capture clinical progression and manifestations. Identified pathways can serve as an entry point for further diagnostic and therapeutic discovery in CADASIL and sporadic VCID.

Apart from protein markers, plasma offers a reservoir for extracellular vesicles (EVs), nano- to micro-sized cell-derived lipid fluid filled particles that contain cell origin surface markers with internal cargo involved in normal and pathophysiological cellular communication [19]. Exosomes are the smallest EVs (30–150 nm) that carry proteins, lipids, and RNA that signal and mark the occurrence of cellular changes but are not restricted by the blood brain barrier. EVs provide the unique opportunity to study cell-specific molecular changes in living organisms and, thus, constitute a form of “liquid biopsy.” Dr. Frank C. Barone investigated exosomal RNA as a potential novel biomarker in mouse ischemic stroke. Exosomes are released from brain-infiltrating inflammatory and vascular endothelial cells in stroke and can be enriched using cell of origin surface molecules using a newly developed microfluidics system. Dr. Barone’s lab described the development of a mouse stroke translational platform of differential gene expression (DGE) to discover new RNA biomarkers and mechanisms. This platform enables comparison to human stroke, through identification of DGE changes in circulating and brain cells from their released exosomes in evolving brain injury. Plasma exosomes are enriched using the following surface markers: (1) CD15, to capture changes in neutrophils, the earliest post-stroke infiltrating immune cells, and (2) VCAM-1, to capture endothelial cell activation and BBB injury as early events in the brain post-stroke. By combining data from exosomes with developing brain spatial transcriptomics in mouse brain sections, the aim is to provide information on markers and localization of activated leukocytes and vascular cells, and to expand this research to brain glia, neurons, and white matter markers and mechanisms in brain hypoperfusion, neurodegeneration, and dementia.

Stroke and dementia frequently co-exist in aging populations, with overlapping risk factors and cumulative effects on cognitive decline. These conditions share age as their strongest risk factor and demonstrate significant sexual dimorphism, with females showing higher lifetime risk for both conditions. When co-occurring, these pathologies interact synergistically to accelerate cognitive deterioration, as evidenced by the Nun’s Study showing that dementia patients with AD pathology who also had cortical infarcts had substantially higher rates of cognitive impairment. While microglia play crucial roles in both conditions as core pathological features, their diverse activation phenotypes present significant challenges for therapeutic development. Current methods for measuring microglial activation, such as cerebral spinal fluid sampling and positron emission tomography imaging, are either invasive or limited by cost and accessibility. Dr. Shawn N. Whitehead’s lab studies circulating microglial extracellular vesicles (MEVs) and their cargo as blood-based biomarkers capable of discriminating between AD pathology and stroke pathology. In order to examine the common and unique MEV profiles in these disorders, aged wildtype and APP/PS1 transgenic rats were used to develop experimental models of aging, AD, stroke, and comorbid AD/stroke. APP/PS1 rats develop age-dependent amyloid-beta deposition and tau pathology, while focal subcortical strokes were induced using endothelin-1 injections into the striatum. Distinct microglial phenotypes and their corresponding MEV markers were identified. These markers were validated using high throughput nanoflow cytometry, which allowed detection of MEVs in as little as 10 µl of the blood plasma. Preliminary analyses focused on TMEM119-positive MEVs containing specific cargo proteins. In future work using this approach, the utility of circulating MEVs as a novel diagnostic tool for monitoring brain inflammation and predicting cognitive decline will be further investigated. The development of this blood-based platform could facilitate early detection of dementia risk and guide therapeutic interventions in patients with AD pathology, stroke, or both conditions.

Peripherally circulating biomarkers and cargo proteins from brain-derived extracellular vesicles (BDEVs) in blood have been shown to track AD pathology progression, predicting cognitive decline and amyloid burden among cognitively normal and mild cognitive impairment (MCI) individuals. However, these biomarkers have yet to be fully explored in saliva. Novel findings presented from Dr. Charisse N. Winston assessed the feasibility and practicality of isolating plasma and saliva BDEVs to identify early predictors of disease pathogenesis and cognitive decline among healthy controls, individuals with MCI, and other age-related dementias. Saliva EVs were extracted and precipitated using a polymer-based isolation method (ExoQuick), and enriched against neuronal (L1CAM, NRXN), astrocyte (GLAST), microglial (TMEM119), and oligodendrocyte (P1P1) sources using magnetic immunocapture and fluorescence-activated cell sorting (FACS). Tetraspanin profiling, super-resolution imaging (ONI nanoimager), and immunoassay were used to characterize EVs by size, shape, and cellular origin. Concentrations of proinflammatory and ATN-related (amyloid, tau, neurodegeneration) cargo proteins were quantified using human-specific ELISAs and MSD immunoassay (V-PLEX Proinflammatory Panel 1). Preliminarily, her study determined that preanalytical storage conditions (room temperature, 4 °C or − 20 °C) and cellular origin significantly impacted EV cargo levels of pro-inflammatory and ATN biomarkers. Interestingly, significant TDP-43 accumulation was exclusive to saliva astrocyte EVs. These findings demonstrate the feasibility and practicality of isolating BDEVs from saliva. Moreover, these findings suggest that preanalytical storage conditions can impact the biomarker stability and sensitivity of BDEV cargos and require further investigations.

The results presented at this session demonstrated the versatility of biofluids as reservoirs of markers that can capture clinical, prognostic, and neuropathological aspects of VCID. Novel methods, such as cell-specific EV isolation, enable precise understanding of disease pathophysiology at the cellular level. The use of such markers alongside established biomarkers of neurodegeneration and AD pathology in the future may allow the distinction of VCID from other causes of cognitive decline and, therefore, appropriate therapeutic interventions.

#### Imaging biomarkers

This workshop section opened with a presentation by Dr. Hanzhang Lu on the possible use of MRI oxygen extraction fraction (OEF) signal as a biomarker for VCID. Microstructural damages using diffusion MRI have also demonstrated considerable potential. A recent trend is to move the biomarker development to earlier and potentially more sensitive cerebrovascular imaging assessments. Brain perfusion and oxygenation are two important physiological parameters of vascular health. Dysfunction in small vessels is expected to affect perfusion. MRI methods can measure brain perfusion. However, perfusion can also be affected by reduced brain metabolism independent of vascular function. Oxygen extraction fraction (OEF) reflects the balance between oxygen delivery and consumption. In a longitudinal study of aging, OEF was found the increase over time [20–22]. The increase in OEF was more prominent in individuals with high vascular risks compared to those with low vascular risks and was associated with progression of vascular risks and the growth in WMH volume. OEF change was not related to CSF markers of AD pathology or their progression. Longitudinal OEF change in older adults is primarily related to vascular pathology. Additionally, unlike CBF which can be reduced by both AD and vascular pathology, OEF appears to be differentially affected in these conditions in that OEF is elevated by vascular pathology but diminished by AD pathology [23].

In another application of MRI technology, Dr. Liu presented on how infra-slow (< 0.1 Hz) oscillatory global brain activity measured by human fMRI, monkey ECoG, and mouse neuronal recordings is closely coupled with CSF flow, as measured through fMRI inflow effect. Infra-slow activity is particularly evident during light sleep, and its close links to CSF flow dynamics may have a role in perivascular clearance of waste products. Dr. Liu and his group found that in neurodegenerative disease, particularly early stages of AD, there is a reduction in this infra-slow activity and its decreased coupling with CSF flow, which they correlate with various AD risk factors and pathologies, including cognitive function. In particular, the coupling strength of this global brain activity with CSF flow is correlated with the accumulation of amyloid-beta (Aβ) and tau. One hypothesis was that CSF flow was disrupted when AD pathology was present because of cerebral amyloid angiopathy causing a blockage of the perivascular spaces and glymphatic system.

The importance of enlarged perivascular spaces both in dementia pathophysiology and as a possible imaging biomarker was explored in presentations by Dr. Konstantinos Arfanakis and Dr Joel Ramirez. Dr. Arfanakis identified that enlarged perivascular spaces (EPVS) are common in older adults, but their neuropathologic correlates are unclear because most work to date has relied on visual rating scales and/or clinical cohorts [24–28]. He presented a deep learning model for automatic segmentation, localization, and quantification of EPVS in ex vivo brain MRI and then used this model to investigate the neuropathologic, clinical, and cognitive correlates of EPVS in 817 community-based older adults that underwent autopsy [29, 30]. The new method exhibited high sensitivity in detecting EPVS as small as 3 mm^3^, good segmentation accuracy and consistency. Most EPVS were located in the frontal lobe, but the highest density of EPVS was observed in the basal ganglia. The total number of EPVS in the cerebrum as well as in the frontal lobe were independently associated with infarcts, while temporal and occipital lobe EPVS were associated with cerebral amyloid angiopathy (CAA) severity. These findings suggest that EPVS share neurobiological pathways with infarcts and CAA and that these pathways are dependent on location within the brain. Basal ganglia EPVS were independently associated with hypertension, providing important new leads on the role of these vascular risk factors in the condition of the glymphatic system. Finally, EPVS were associated with lower level of cognition above and beyond the effects of neuropathologies, clinical variables, and demographics, suggesting that EPVS represent additional brain anomalies contributing to lower cognitive function [31]. Dr. Ramirez also acknowledged that MRI-visible perivascular spaces (mPVS) may play a crucial role in sleep-driven glymphatic clearance of brain metabolic waste, similar to lymphatic vessels in peripheral tissues [32]. In contrast to previous studies that have demonstrated mPVS burden in the white matter/centrum semiovale is associated with AD pathology and CAA pathology, in Parkinson’s disease, large mPVS (> 3 mm) in the basal ganglia are associated with motor symptoms and motor complications, while smaller mPVS (≤ 3 mm) are associated with motor and nonmotor aspects of experiences in daily living [33]. Lastly, complimenting Dr. Arfanakis’ work relating mPVS and cognition, Dr. Ramirez found in patients with neurodegenerative disease that the effect of mPVS on speeded executive function was mediated by the presence of elevated glial fibrillary acidic protein (GFAP), a blood-based biomarker of inflammation, and of white matter hyperintensities [34].

In sum, this section of the workshop identified that oxygen extraction fraction may be a more specific marker of VCID, that the uncoupling of CSF flow and infra-slow activity may help us understand dementia pathology, and that enlarged perivascular spaces may be one of a suite of imaging biomarkers for VCID.

### Risk and disease management

In the final workshop section, a diverse but connected group of presentations challenged current understandings of dementia risk factors, ranging from domestic violence, Down syndrome, and hypertension. Dr. Cheryl L. Wellington identified that one in three women worldwide experience intimate partner violence (IPV), 92% of whom report head impacts and/or strangulation that raise suspicion of brain injury (BI). Despite the high prevalence, there is insufficient understanding about long-term risks, including Alzheimer’s disease and related dementias (AD/ADRD); knowledge of IPV neuropathology is scant, with vascular pathologies rather than proteinopathies observed in most cases. Dr. Wellington discussed an in-progress prospective observational study aiming to enroll 500 acute/subacute and 100 chronic survivors of IPV. The study aims to use a proteomic platform to assess plasma biomarkers as diagnostic tools, to develop a living clinical practice guideline for IPV-BI, and create trauma-informed guidance for AD/ADRD researchers to ask about IPV in clinical history. She discussed some of her team’s emerging animal models of non-fatal strangulation to investigate inflicted hypoxic-ischemic injury as a potential VCID component.

In a further presentation on another crucial but underserved population who experiences dementia, Dr. Elizabeth Head presented research for people with Down syndrome (DS) who have a 90% lifetime risk of developing dementia. Due to an extra copy of chromosome 21 and associated triplication of the amyloid precursor protein gene, almost all people with DS develop AD neuropathology by 40 years of age. Interestingly, despite being protected from vascular risk factors, such as hypertension and atherosclerosis, the same overexpression of the beta-amyloid precursor protein leads to vascular accumulation of beta-amyloid as cerebral amyloid angiopathy (CAA). Indeed, the extent of CAA in DS exceeds that observed in late-onset AD dementia. Increased CAA is associated with microhemorrhages and reduced cerebral blood flow (CBF) as measured using magnetic resonance imaging and arterial spin labeling. Whereas previously, dementia in DS was thought to be associated with a “pure” AD-like pathology, it is clear that cerebrovascular dysfunction is a key contributor to pathological brain aging.

While those with DS do not commonly experience hypertension, hypertension is the most common risk factor for dementia and was the focus of the presentation by Dr. Matthew Lennon. He identified that it affects 1.3 billion persons worldwide, is the most prevalent risk factor for dementia, and in the majority of cases, it is either undiagnosed or uncontrolled [35]. He presented on a number of studies used to investigate the hypertension-dementia relationship, including a large consortium of longitudinal cohort studies and the UK Biobank. In a study utilizing the Cohort Studies of Memory in an International Consortium (COSMIC) group (*n* studies = 17 and *n* = 34,519), individuals with untreated hypertension had a 26% (*p* = 0.02) and 42% (*p* = 0.0135) increased risk of AD dementia compared with individuals with treated hypertension [36, 37]. Those without hypertension had no significantly different risk compared to those with treated hypertension, whereas compared to those with untreated hypertension, they had a 42% (*p* = 0.001) and 36% (*p* = 0.04) lower risk of dementia and AD dementia, respectively. In the UK Biobank (*n* = 448 575), using a Mendelian randomization approach, it was found that genetic propensity for high SBP had an approximately linear association with worsened fluid intelligence (*p* = 0.0018) but better reaction time (*p* < 0.0001) [38]. He concluded with the ideas that untreated high blood pressure increases overall dementia and AD dementia risk throughout late life, but may differentially affect cognitive domains. Providing clear education to the public that hypertension is a significant risk for dementia may change the way that clinicians and patients screen for and manage hypertension.

Hypertension, among other risk factors, increases risk for cerebral ischemia and cerebral small vessel disease. Dr. Aravind Ganesh presented on current trials of remote ischemic conditioning (RIC) to prevent progression of cSVD and reduce the impact of cerebral ischemia. RIC [39, 40] involves placing a body part, generally the arm, under ischemic conditions for several minutes at a time. It is hypothesized that such intervention may promote proteomic and transcriptional changes that are neuroprotective, making individuals more resilient to ischaemic damage. Dr. Ganesh and his team studied the safety/tolerability of RIC in patients with cSVD in TRIC-VCI (NCT04109963)—a prospective, open-label, randomized-controlled trial with blinded endpoint assessment and 14-day run-in period, after which participants were randomized to once-daily or twice-daily RIC for 30-days [41]. Among 22 patients who completed the run-in phase and were randomized, 7/11 (63.6%) in the once-daily RIC arm completed ≥ 80% of sessions versus 10/11 (90.9%) in the twice-daily arm. In parallel, they found that just 2 weeks of RIC in mice drastically modifies the central nervous system’s proteomic and transcriptional landscape as seen in blood and spinal cord homogenates, with upregulation of several neuroprotective pathways. A current pilot clinical trial of a compact, automated RIC device, TRIC-SVD (NCT05967728) will enroll 24 patients with cSVD and seek to further validate the blood biomarkers of interest identified in their prior work.

## New information and future Albert White Matter and Cognition Conferences

This year’s Albert Research Institute workshop provided insights into diverse features of the VCID, including the following:

- Vascular pathology is present in the majority of dementia cases.
- VCID is of special interest to international neuroscientific research programs; for instance, over 150 NIH awards and 21 targeted funding announcements aim to develop effective biomarkers, targets, and treatments for dementia.
- Microglia show pronounced senescence signatures in aging and when AD pathology is present.
- Senescence-associated secretory factors represent potential interventional targets for neurodegenerative diseases.
- Retinal changes offer promising biomarkers for VCID, particularly cerebral small vessel disease, with HHcy-induced models revealing specific visual deficits and vascular alterations.
- Retinal biomarkers reflect neuroinflammatory and vascular gene expression changes, informing vision-dependent cognitive testing interpretations.
- AI frameworks using multimodal data can enhance differentiation between AD dementia and non-Alzheimer’s dementias, including vascular dementia, as well as among different dementia-associated pathologies.
- Proteomic studies have identified plasma proteins, including oligodendrocyte myelin glycoprotein (OMG) and neuronal pentraxin receptor (NPTXR), related to white matter hyperintensity volume, motoric cognitive risk, and vascular dementia, with replication in external cohorts.
- Plasma proteomic signatures have been identified in CADASIL indicating dysregulations in fibrosis, angiogenesis, and metabolism, with unique signatures across clinical stages.
- Markers of mural cell loss, fibrosis-coagulation, and T cell activation are associated with disease progression in CADASIL, suggesting targets for therapeutic discovery. The development of a transcript biomarker platform using the mouse ischemic stroke will be used to identify differentially expressed transcripts from enriched exosomes as post-stroke markers of leukocyte and vascular endothelial cell changes and are being validated with human data and spatial transcriptomics to investigate new mechanisms and targets for stroke-related brain injury.
- A method has been developed to use circulating microglial extracellular vesicles (MEVs) as blood-based biomarkers for differentiating between AD and stroke pathologies.
- The utility of MEVs in monitoring brain inflammation and predicting cognitive decline is being investigated to facilitate early detection and guide therapeutic interventions.
- Plasma and saliva brain-derived extracellular vesicles (BDEVs) offer a feasible method to identify early predictors of disease pathogenesis and cognitive decline.
- Perfusion reflects blood supply to the brain, while oxygen extraction fraction (OEF) balances oxygen delivery and consumption; both can be measured using MRI.
- OEF changes in older adults are associated with vascular pathology and progression of vascular risks, highlighting its potential as a VCID biomarker.
- Infra-slow (< 0.1 Hz) global brain activity is enhanced during light sleep and is coupled with CSF flow, potentially aiding in brain waste clearance.
- A deep learning model has been developed to segment and quantify EPVS in ex vivo brain MRI, identifying neurobiological pathways shared with infarcts and CAA.
- EPVS are linked to brain waste clearance and are associated with cerebral small vessel disease, hypertension, diabetes, poor sleep, and cognitive decline in clinical-radiological and clinical-pathological studies.
- EPVS burden correlates with executive function and white matter hyperintensities, highlighting its potential as a biomarker for vascular health and dementia.
- Decreases in cerebral blood flow (CBF) with aging in DS are linked to cognitive decline, highlighting the importance of cerebrovascular pathology in designing clinical trials for this population.
- High prevalence of head impacts and strangulation in intimate partner violence (IPV) raises suspicion of brain injury (BI). The size of the contribution of IPV to vascular disease and dementia remains an open question.
- Ongoing studies aim to improve the diagnosis of IPV-BI using blood-based biomarkers and animal models to investigate hypoxic-ischemic injury.
- Untreated hypertension increases the risk of dementia, including AD dementia with AD pathology, emphasizing the importance of managing blood pressure.
- Genetic predisposition to high systolic blood pressure is associated with worsened fluid intelligence but better reaction time, indicating possible differential effects on cognitive domains.
- Remote ischemic conditioning (RIC) shows promise in preventing cerebral small vessel disease (cSVD) progression, with upregulation of neuroprotective pathways; candidate treatment-related biomarkers like VCAM1 and FABP3 have been identified.
- Future foci for the Albert Institute Conference will include the following:
  - Molecular and imaging biophenotyping of VCID to better subtype, prognosticate, and treat disease presentations
  - Weighing the relative burden of vascular, Alzheimer’s, and other neurodegenerative biomarkers to develop rational, personalized, multi-mechanism treatment of cognitive disorders
  - Better understanding trajectories of decline, treatment responsiveness, and possibility of reversal of the underlying VCID pathologies to better inform clinical management

## Discussion and conclusions

The data presented during the workshop highlighted the widespread prevalence of covert and symptomatic VCID in the population and the emergence of novel prognostic and diagnostic modalities. The ensuing round-table discussion was an opportunity to identify unifying concepts across different lines of work and address open questions. A prominent challenge in designing biomarkers for VCID stems from the heterogeneity of the term, which encompasses a wide range of conditions with variable pathophysiology. As a result, each biomarker needs to be assessed as to which underlying processes it quantifies. Some biomarkers may capture processes that are common across types of VCID. Therefore, interpretation of VCID biomarkers needs to be well-defined before they become incorporated in clinical trials and clinical practice. A thorough understanding about what a diagnosis that falls under the VCID scientific framework, not limited to vascular dementia, AD dementia, CADASIL, or post-stroke dementia, would enable better communication between clinicians and patients and provide preventive or management options to patients that would otherwise not be available. In parallel, participants acknowledged that pure pathological entities, including cerebrovascular changes, are rare in clinical practice. This is in contrast to the prevailing notion in research which assigns clinical syndromes to specific disease categories with specific pathologies may or may not be present in a given individual. For example, during the presentations, the term AD was used interchangeably to denote clinical, biomarker-based, and pathology-proven forms of the disease. In reality, patients may present with specific clinical syndromes which do not exclude the co-occurrence and synergistic effects of multiple pathologies. Efforts must be undertaken to better define VCID as a driver or contributor to clinical outcomes.

The initiative led by the Albert Research Institute for White Matter and Cognition provided an opportunity for the advancement of the open questions surrounding diagnoses that fall under the VCID scientific framework. By examining different approaches during the course of two days, participants were able to engage regarding state-of-the-art biomarker tools and envision future directions by incorporating multidisciplinary viewpoints.

## Data Availability

This is not applicable as we have not reported an original research study.
